# End of Volume Fifth

**Published:** 1850-05

**Authors:** 


*End of Volume Fifth.*—With the present number ends the *fifth* volume of the *Buffalo Medical Journal.* In connection with this announcement it is, perhaps, not too great a stretch of presumption to suppose that it may afford gratification to some of our readers to know something of the rise and progress of the work which receives their support, and the circumstances under which it is to be continued. We may assume that a few words on these matters may not be unacceptable, the more, because we cannot be accused of often obtruding the affairs of the Journal upon the attention of its patrons.

Five years ago we were induced to undertake the management of a monthly Medical Journal of 24 pages, at the instance of a few professional friends. We entered upon our editorial duties with the impression, (which we believe is not unusual with the inexperienced,) that they would serve to fill up some spare moments, and furnish a kind of pastime to relieve the laborious duties of medical practice. To any one who has been betrayed by similar anticipations, it is needless to say that they were soon found to be delusions. The task of providing more or less editorial matter for each number; preparing contributions for the compositor; examining journals for selections, and new publications for notice or reviewal; together with the reading and revision of proof, which has always devolved entirely on the editor, was discovered, so soon as the novelty of a new occupation had abated, to be little calculated for relaxation, but, on the contrary, to involve not only a new accession of labor, but labor, not a small portion of which is of that kind called drudgery. Nevertheless, we did not allow a realization of the toils of editorship, unexpected, and in many respects unprofitable, as they were, to deter us from the prosecution of the undertaking to which we were unwittingly committed. Having put our hand to the plow, we felt no disposition to turn back. Providentially or otherwise, the newly born bantling was on our hands, and not only could not with propriety be abandoned, but we felt bound to fulfil, in so far as practicable, the obligations incident to our *responsibility,* by exerting ourselves, with parental concern, to promote its growth and prosperity. With these sentiments, at the expiration of the first volume, the size and character of the work were materially changed. The number of pages was increased to 64 (monthly), and the contents of each number distributed in three departments, as at present. The only alterations in the internal arrangement of the work since the commencement of the second volume, has been a change of type from *small pica* to *long primer,* the latter giving an improved typographical appearance, and more matter in an equal space.

For the first three years, the Journal was published at a considerable pecuniary sacrifice on the part of editor and publishers. To the latter our thanks are due for their liberality in continuing the publication under such circumstances, which they were led to do by their interest in the enterprise, and the belief that it would in the end succeed. We are happy to say that these expectations have proved correct. The patronage of the work has continually increased, without much exertions having been made by travelling agents, etc., to extend its circulation; and it is now amply sufficent to render its publication an object with its proprietors. Its stability, therefore, in so far as this depends on the list of subscribers, is amply secured.

The patronage of a medical periodical is perhaps a fair test of its acceptableness. We may be permitted, however, to say, that, in addition to this, other evidence of the favor with which the work has been regarded by the medical profession, has afforded no small degree of encouragement and gratification. We may thus allude to the flattering notices which it has received, both of a public and private nature, without the charge of egotism or indelicacy, inasmuch as we are fully sensible that whatever merit the Journal possesses, is, in a great measure, due to the valuable contributions which it has elicited from its correspondents; and in making this acknowledgement (which we do in all sincerity), we would have it considered as the measure of our sense of personal obligation to those who have contributed articles for our pages. More especially should we disclaim any assumption to the prejudice of our colleagues of the Medical Faculty of the University of Buffalo, to whom, conjointly with the editor, belongs whatever credit may be attached to having originated and continued the work.

It may seem to some that the present age of our Journal embraces too small a cycle to furnish a proper occasion for a historical sketch of its rise and progress—it may be said to be still in its infancy, and that formal retrospections should be deferred to a later period in its career. There may be some justness in such a criticism, but there are few things relating to human life that are to be measured by an abstract rule—every thing is to be considered relatively. Youthful as we are (i. e. as a journalist,) during the five years of our editorial service we have seen many changes in our contemporaries. Several journals during that period have been discontinued; some have commenced and ended a brief career; and we believe we are correct in stating that of the various medical publications which were in existence when the first number of the Journal was issued, all but two of those remaining at the present time, have passed into the hands of new, and, in some instances, successive editors. Under such circumstances, we certainly think we have some claim to be considered one of the *senior* members of the fraternity. But lest we should be suspected of becoming garrulous in our old age, we will say no more of the *past* and *present*, and conclude with a few desultory remarks respecting the *future*.

The *sixth* volume of the *Buffalo Medical Journal* will commence with the No. for June, (proximo) and will continue in the hands of the present editor and publishers. No changes in the style of its publication, terms, etc., are at present contemplated.

The "*original department*," as heretofore, will be open to contributions on medical subjects, and we shall be glad to receive articles from an increased circle of correspondents. We would cordially invite our brethren to furnish reports of cases, results of experience, and reflections upon any of the numerous topics that concern, directly or indirectly, the interests of the profession, or the progress of medical science, pledging ourselves to endeavor, in so far as in us lies, to do justice to whatever may be placed at our disposal. They to whose co-operation the work is indebted, in a great measure, for whatever interest and usefulness it now possesses, will, as we trust, continue their valued assistance, which, we doubt not, is appreci ated by all our readers as fully as by ourselves.

In the "*eclectic department*," we shall continue to cull from contemporaneous journals, and new publications of all kinds, such extracts as shall appear to us most deserving of selection, giving preference always to those which are of practical utility, but not overlooking, altogether, what we may judge will be acceptable to medical readers, albeit not having direct, important relations with the duties of the medical practitioner. It will be with us, as heretofore, a prime object to gather, in this department, choice contributions by *American* writers. We feel this to be our duty, believing that *justice*, not less than *charity*, should begin at home. But it is a pleasure not less than a duty with us to pursue this course. While we hold that *science*, as well as letters, is a *republic*, occupying a position above political institutions, and rising superior to sectional prejudices, we believe that a certain degree of nationality of feeling is by no means inconsistent with such a doctrine, and is much to be desired. We think, moreover, that, in the province of medicine, there is, in this country, a deficiency of that patriotic spirit which is infused into other pursuits, and we believe, with a view to progress and improvement, as well as a national position, it is important that this spirit be fostered and increased. Is there any reason why the freedom, the energy, and the talents entering into the *American character*, should be less conspicuously displayed here, than elsewhere? We are not only ready to answer this question in the negative, but we do not hesitate to avow a belief that the medical profession of this country, at this moment, embraces an amount of ability adequate to accomplish as much for science and humanity, and its own honor, as that of any other nation. If we err in this opinion, it is not a less venial error than an under estimate of the medical profession of our own country, more especially since the former tends to stimulate, as much as does the latter to repress, a generous spirit of emulation, from which no evil, but great good may spring. To return from this digression, we deem it not only proper, but important, for the medical journalist to endeavor, first of all, to prize and promote the science and literature of his own country, resisting at the same time a degree of national bias which would lead him to overlook, or depreciate aught that originates in a foreign clime. Our aim as a journalist, is to do what little we can to carry out these views.

In the "Editorial Department" the qualities which we wish might characterize our articles are, candor, impartiality and independence. We may take this occasion to say that, as an editor, we are entirely untrammeled. We are responsible to no one, nor are we subject to the dictation of any. We are under no obligations which should exercise any control over us. We continue the Journal chiefly because, persuading ourselves of its utility, we think it is our duty to continue it; and we shall be well satisfied to give it up whenever we think otherwise. We place no expectations of reputation or pecuniary profit upon it, and have nothing to loose by it, so that it be reputably conducted. There are, then, no reasons why we should not always write with freedom and boldness, since there is nothing to restrain or guide us aside from the dictates of our own judgment and taste. But we are too often rallied by our familiar friends on the subject, not to be aware that there is much room for improvement in this particular, as doubtless in many other respects. We confess that we had rather err in over-consideration for the feelings and opinions of professional brethren, than to expose ourselves to the consciousness of arrogance or rudeness. Moreover, we are anxious, above all things, to live in harmony and peace. Quietness has for us more charms than excitement, and contentions are our abhorrence. Such, we know, are not the feelings best calculated to secure raciness and interest for the pages of a periodical; but we would much prefer to divest ourselves of the office of an editor, than to compromise the conditions of personal comfort. Nevertheless, with respect to events transpiring in the medical world which are properly subjects of open comment, the conflicting opinions of professional brethren, medical publications, etc., it is obviously the duty, as well as privilege, of an editor, to speak his mind plainly and without reserve. We admit that it is sometimes better to do this with the risk of error, (which is no reproach, since all are fallible,) than to yield too much to maxims of over-prudence. If we may judge of the future by our present intentions, we shall hereafter act more in accordance with the convictions just expressed.—But we have written quite enough on the occasion of entering upon a new volume, and we will therefore abruptly conclude by assuring the reader that if he will pardon this exercise of the editorial license to indulge a little loquacity on *our* affairs, we will reserve another similar trial of his patience for some more extraordinary epock in the history of the *Buffalo Medical Journal.*

